# Differences in right ventricular function and response to targeted therapy between patients with IPAH and PAH-CHD

**DOI:** 10.3389/fphar.2023.1124746

**Published:** 2023-02-13

**Authors:** Tengteng Zhu, Panyun Wu, Zhen Tan, Quan Jin, Yusi Chen, Lian Li, Zewei Chen, Yirui Tang, Jiang Li, Zhenfei Fang

**Affiliations:** Department of Cardiovascular Medicine, The Second Xiangya Hospital, Central South University, Changsha, Hunan, China

**Keywords:** congenital heart disease, pulmonary arterial hypertension, pulmonary hypertension, right ventricle, right ventricular function, targeted therapy, treatment

## Abstract

**Background and aims:** Pulmonary arterial hypertension (PAH) is a chronic pulmonary vascular disorder characterized by elevated pulmonary vascular resistance (PVR) and pulmonary arterial pressure (PAP). Right heart failure is a life-threatening complication of PAH and predicts a poor prognosis. PAH associated with congenital heart disease (PAH-CHD) and idiopathic PAH (IPAH) are two prevalent PAH subtypes in China. In this section, we set out to explore baseline right ventricular (RV) function and its response to targeted agents between IPAH and PAH-CHD.

**Methods and results:** Consecutive patients diagnosed with IPAH or PAH-CHD by right heart catheterization (RHC) in the Second Xiangya Hospital from November 2011 to June 2020 were included. All patients received PAH-targeted therapy and the RV function was assessed by echocardiography at baseline and during follow-up. A total of 303 patients (age, 36.23 ± 13.10 years; women, 213 (70.3%); mean PAP [mPAP], 63.54 ± 16.12 mmHg; PVR, 14.74 ± 7.61 WU) with IPAH (*n* = 121) or PAH-CHD (*n* = 182) were included in this study. Compared with PAH-CHD, patients with IPAH had worse baseline RV function. As of the latest follow-up, forty-nine patients with IPAH and six patients with PAH-CHD died. Kaplan-Meier analyses showed better survival in PAH-CHD versus IPAH. After PAH-targeted therapy, patients with IPAH had less improvement in 6 MWD, World Health Organization functional class, and RV functional parameters compared with patients with PAH-CHD.

**Conclusion:** Compared with patients with PAH-CHD, patients with IPAH had worse baseline RV function, unfavourable prognosis, and inadequate response to targeted treatment.

## 1 Introduction

Pulmonary arterial hypertension (PAH) is a chronic pulmonary vascular disease characterized by elevated pulmonary vascular resistance (PVR) and pulmonary arterial (PA) pressure (PAP). Currently, the incidence and prevalence of PAH are approximately 5/million and 25/million, respectively (Hoeper et al., 2016). According to the updated 2022 European Society of Cardiology/European Respiratory Society (ESC/ERC) guideline, PAH is pre-capillary pulmonary hypertension defined as mean PAP (mPAP) > 20 mmHg, PVR >2Wood Units and PA wedge pressure (PAWP) ≤15 mmHg as measured by right heart catheterization (RHC) (Humbert et al., 2022). Clinically, PAH is divided into the following subgroups based on etiology, including idiopathic PAH (IPAH), heritable PAH, drug- and toxin-induced PAH, PAH associated with other conditions [such as connective tissue disease (CTD), congenital heart disease (CHD)], PAH with features of venous/capillaries involvement, and persistent PAH of the newborn (Humbert et al., 2022). At present, the pathogenesis of PAH remains unclear, and pulmonary arterial remodeling is an important pathological hallmark. Similar to malignancy, patients with PAH have an extremely poor prognosis (Ruopp and Cockrill, 2022). Previous studies demonstrated that the median survival of treatment-naive patients with IPAH was only 2.8 years, and the 1- and 5-years survival rates were 68% and 34%, respectively (D'Alonzo et al., 1991). During the past few decades, advances in understanding of the pathogenesis of PAH have led to significant progress in PAH-targeted medications, including phosphodiesterase-5 inhibitors (PDE-5i), endothelin receptor antagonists (ERAs), prostacyclin analogues and prostacyclin receptor agonists (PGI2), and soluble guanylate cyclase stimulators (sGCs). Notably, although targeted drugs improve the symptoms and haemodynamics, the 5-years survival rate of PAH has only increased to over 60% (Farber et al., 2015), which has not yet been satisfied.

Elevated PVR and PAP increase the right ventricular (RV) afterload and promote adaptive or non-adaptive RV remodeling, which triggers RV dysfunction and right heart failure. Studies have revealed that RV function is closely related to functional class and prognosis in patients with PAH, and deteriorating cardiac function portends a poor prognosis. To our knowledge, there were significant survival differences among different subgroups of PAH. The survival of patients with IPAH was inferior to that of patients with PAH associated with CHD (PAH-CHD) (Manes et al., 2014), but superior to that of patients with CTD-associated PAH (CTD-PAH) (Chung et al., 2010; Chung et al., 2014; Ramjug et al., 2017). In addition, significant survival differences were also observed between different subtypes within the same subgroup. Chung and colleague confirmed that patients with systemic sclerosis (SSc)-associated PAH (SSc-PAH) have a higher mortality rate than those with CTD other than SSc-associated PAH (Chung et al., 2010). In the PAH-CHD cohort, when compared with patients with Eisenmenger syndrome (ES) or PAH-CHD associated with systemic-to-pulmonary shunts (SPs), patients with small defects (SDs) or PAH after defect correction (CDs) had the worse prognosis (Manes et al., 2014). Previous studies have shown that diverse RV function and response to PAH-targeted therapy are associated with differences in prognosis between patients with CTD-PAH and patients with IPAH (Rhee et al., 2015; Ramjug et al., 2017). In China, PAH-CHD and IPAH are two prevalent types of PAH, and their baseline right heart function and response to targeted drugs are less studied. Given that RV dysfunction are closely associated with poor prognosis, we intend to explore baseline RV function and its response to targeted agents between IPAH and PAH-CHD.

## 2 Patients and methods

### 2.1 Study designs and subjects

We retrospectively collected and studied adult treatment-naive patients with IPAH or PAH-CHD who were admitted to the Second Xiangya Hospital from November 2011 to June 2020. All recruited patients with IPAH or PAH-CHD met the following diagnostic criteria: mPAP ≥ 25 mmHg, PVR >3 WU and PAWP ≤15 mmHg during resting RHC (Galiè et al., 2016). Except for IPAH and PAH-CHD, patients with other types of PAH or PH belonging to groups 2–5 were also excluded. All recruited patients were prescribed PAH-specific therapy based on multiparameter risk stratification of contemporaneous guidelines, and cardiac function and haemodynamics parameters were assessed by means of echocardiography. In the 2022 ESC/ERS guideline for pulmonary hypertension, it was recommended to use a three-strata risk-assessment model to classify patients as low, intermediate, or high risk at initial assessment, and to use a four-strata model to classify patients as low, intermediate-low, intermediate-high, or high risk during follow-up (Humbert et al., 2022). Risk stratification can help guides treatment decisions in patients with PAH. Briefly, initial monotherapy with PDE-5i or ERAs was recommended for patients with PAH and cardiopulmonary comorbidities. In patients with PAH without cardiopulmonary complications, initial dual combination therapy of ERAs and PDE-5i was recommended for patients with low- or intermediate-risk of death, and triple combination therapy of ERAs, PDE-5i, and prostacyclin analogue is recommended for patients with high-risk of death. At follow-up, patients who reach low-risk status continued the initial regimen, and patients with medium-low risk were suggested to add prostacyclin receptor agonist or replace PDE-5i to sGC. In addition, intravenous or subcutaneous prostacyclin or evaluation for lung transplantation was recommended for patients who had insufficient treatment response and were still at intermediate-high or even high risk. PAH-specific medications in this study included PDE-5i (e.g., sildenafifil and tadalafifil), ERAs (e.g., bosentan, ambrisentan, and macitentan), prostacyclin analogues (e.g., intravenous or subcutaneous treprostinil, intravenous epoprostenol, and inhaled iloprost), prostacyclin receptor agonists (slexipag), and sGCs (e.g., riociguat). The study was approved by the Ethics Committee of the Second Xiangya Hospital.

### 2.2 Date collections

The following electronic medical record data were collected: age, gender, heart rate (HR), arterial oxygen saturation (SaO_2_), 6-min walking distance (6 MWD), World Health Organization functional class (WHO FC), and application of PAH-targeted drugs.

Transthoracic echocardiography (TTE) was performed according to the guidelines (Mitchell et al., 2019), and the following parameters were recorded: tricuspid regurgitation velocity (TRV, m/s), RV systolic pressure (RVSP), PA systolic pressure (PASP), left ventricular ejection fraction (LVEF), tricuspid annular plane systolic excursion (TAPSE), RV fractional area changes (RVFAC), and pericardial effusion. PASP is equal to RVSP in the absence of right ventricular outflow tract and pulmonary artery stenosis. RVSP was estimated according to the simplified Bernoulli’s equation, i.e., 4 × TRV^2^ + right atrial pressure. The TAPSE/PASP ratio was used to assess the RV-PA coupling (Tello et al., 2019).

A haemodynamic assessment was performed by RHC *via* the right femoral vein. Pressure in the right atrium, right ventricle, and pulmonary artery was recorded by fluid-filled catheters connected to pressure transducers. Pulmonary (Qp) and systemic (Qs) flow was calculated by the Fick method (Miranda et al., 2022). PVR was calculated as (mPAP-PAWP) divided by Qp. Cardiac index (CI) was calculated as cardiac output divided by body surface area.

### 2.3 Statistical analysis

In the present study, the follow-up time was defined as the interval from baseline time of RHC diagnosis to 1 November 2022. Continuous variables with a normal or abnormal distribution were expressed as mean ± standard deviation (SD) or median [interquartile range (IQR)], respectively, and independent samples *t*-test or Mann-Whitney *U* test was used to compare continuous variables between two groups. Categorical variables were expressed as frequency (percentage) and chi-square test was used for comparisons between two groups. The overall survival distribution was analysed by the Kaplan-Meier method. Log-rank test was used to compare differences in survival between groups. All statistical analyses were performed by Statistic Package for Social Science (SPSS, version 19; IBM Corp., Armonk, NY, United States) and GraphPad Prism 8 (GraphPad Software, Inc., San Diego, CA, United States). Differences were considered statistically significant when *p*-value <0.05.

## 3 Results

### 3.1 Demographic and clinical characteristics

According to the inclusion and exclusion criteria, a total of 303 adult treatment-naive patients (age, 36.23 ± 13.10 years; women, 213 (70.3%); mPAP, 63.54 ± 16.12 mmHg; PVR, 14.74 ± 7.61 WU) with IPAH (*n* = 121) or PAH-CHD (*n* = 182) were included in this study. The overall follow-up time was 66.04 ± 24.53 months. There was no significant difference in follow-up time between the two cohorts.

Baseline demographic and clinical characteristics, echocardiographic and hemodynamic parameters were presented in Table 1. As illustrated in Table 1, no significant differences were observed in age, gender, HR, WHO FC, and follow-up time in patients with IPAH versus PAH-CHD. Patients with IPAH had higher SaO_2_, NT-ProBNP and worse 6 MWD as compared to patients with PAH-CHD (all *p* < 0.05).

**TABLE 1:** Demographic and clinical characteristics at baseline in IPAH and PAH-CHD.

Variables	Total population (*n* = 303)	IPAH (*n* = 121)	PAH-CHD (*n* = 182)	*p*-value
Female sex, n (%)	213 (70.30)	80 (66.12)	133 (73.08)	0.194
Age, y	36.23 ± 13.10	37.78 ± 13.27	35.20 ± 12.93	0.094
HR, bpm	85.30 ± 13.90	85.64 ± 13.49	85.08 ± 14.21	0.735
SaO_2_, %	93.92 ± 4.03	95.15 ± 3.01	93.10 ± 4.40	<0.001
NT-ProBNP, ng/mL	1017 (325.36, 2813.56)	2059.62 (898.99, 4029.09)	713.56 (223.78, 1403.30)	<0.001
6 MWD, m	380.80 ± 78.61	355.31 ± 92.32	397.75 ± 62.73	<0.001
WHO FC, n (%)				0.621
Ⅰ-Ⅱ	130 (42.90)	54 (44.63)	76 (41.76)	
Ⅲ-Ⅳ	173 (57.10)	67 (55.37)	106 (58.24)	
Echocardiography				
TRV, m/s	4.45 ± 0.72	4.40 ± 0.72	4.48 ± 0.72	0.386
PASP, mmHg*	90.74 ± 25.96	90.08 ± 26.59	91.18 ± 25.59	0.720
LVEF, %	61.69 ± 7.74	61.36 ± 6.08	61.90 ± 8.68	0.527
TAPSE, mm	13.12 ± 2.67	11.98 ± 2.25	13.88 ± 2.66	<0.001
RVFAC, %	29.42 ± 4.51	28.45 ± 4.92	30.06 ± 4.11	0.003
TAPSE/PASP, mm/mmHg	0.16 ± 0.06	0.15 ± 0.06	0.17 ± 0.06	0.009
Pericardial effusion, n (%)	71 (23.43)	49 (40.50)	22 (12.09)	<0.001
Right heart catheterization				
mPAP, mmHg	63.54 ± 16.12	58.31 ± 12.57	67.02 ± 17.28	<0.001
mRVP, mmHg	42.96 ± 11.85	41.77 ± 12.30	43.76 ± 11.50	0.152
mRAP, mmHg	11.27 ± 6.14	12.93 ± 6.15	10.17 ± 5.91	<0.001
PAWP, mmHg	8.62 ± 2.09	8.55 ± 1.71	8.66 ± 2.30	0.635
Qp, L/min	4.46 ± 1.89	3.21 ± 1.17	5.295 ± 1.82	<0.001
Qs, L/min	3.67 ± 1.54	3.21 ± 1.17	3.98 ± 1.67	<0.001
Qp/Qs	1.27 ± 0.50	1	1.45 ± 0.58	<0.001
CI, L/min/m^2^	3.06 ± 1.36	2.09 ± 0.73	3.71 ± 1.30	<0.001
PVR, WU	14.74 ± 7.61	17.69 ± 7.89	12.79 ± 6.75	<0.001
SvO_2_, %	60.75 ± 9.13	57.96 ± 9.90	62.60 ± 8.09	<0.001
Treatment				0.007
Monotherapy	75 (24.75)	20 (16.53)	55 (30.22)	
ERA	43 (61.43)	13 (65)	35 (60)	
PDE-5i	15 (21.43)	5 (25)	10 (20)	
PGI2	12 (17.14)	2 (10)	10 (20)	
Combination therapy	228 (75.25)	101 (83.47)	127 (69.78)	
ERA + PDE-5i	143 (62.72)	55 (54.46)	88 (69.29)	
ERA + PDE-5i + PGI2	38 (16.67)	24 (23.76)	14 (11.02)	
PDE-5i + PGI2	25 (10.96)	14 (13.86)	11 (8.67)	
ERA + PGI2	16 (7.02)	6 (5.94)	10 (7.87)	
ERA + sGCs	6 (2.63)	2 (1.98)	4 (3.15)	
Follow-up (months)	66.04 ± 24.53	60.60 ± 23.52	68.05 ± 23.90	0.222

PAH, pulmonary arterial hypertension; IPAH, idiopathic PAH; CHD, congenital heart disease; PAH-CHD, PAH associated with CHD; HR, heart rate; bpm, beats/min; NT-proBNP, N-terminal pro hormone brain natriuretic peptide; 6 MWD, 6-min walking distance; WHO FC, World Health Organization functional class; TRV, the velocity of tricuspid valve regurgitation; PASP, pulmonary artery systolic pressure; LVEF, left ventricular ejection fraction; TAPSE, tricuspid annular plane systolic excursion; RVFAC, right ventricular fractional area changes; mPAP, mean pulmonary artery pressure; mRVP, mean right ventricular pressure; mRAP, mean right atrial pressure; PAWP, pulmonary artery wedge pressure; Qp, pulmonary artery blood flow; Qs, systemic artery blood flow; CI, cardiac index; PVR, pulmonary vascular resistance; WU, Wood unites; SvO_2_, oxygen saturation of mixed venous blood. ERA, endothelin receptor antagonists; PDE-5i, phosphodiesterase type-5, inhibitors; sGCs, soluble guanylate cyclase stimulators; PGI2, prostacyclin analogues and prostacyclin receptor agonists.

TTE showed no significant differences in baseline LVEF, TRV, and PASP between the two groups. Patients with IPAH had a higher incidence of pericardial effusion, lower TAPSE, lower RVFAC, and worse RV-PA coupling (TAPSE/PASP) than patients with PAH-CHD (all *p* < 0.05). RHC revealed that patients with IPAH had more severe PAH than those with PAH-CHD, with a lower mPAP, CI, and SvO_2_, and higher PVR and mRAP (all *p* < 0.05), without statistical difference in PAWP and mRVP. In summary, IPAH group had worse baseline RV function and more severe hemodynamic status.

### 3.2 Treatment strategy, survival analysis, and treatment response

**FIGURE 1:**
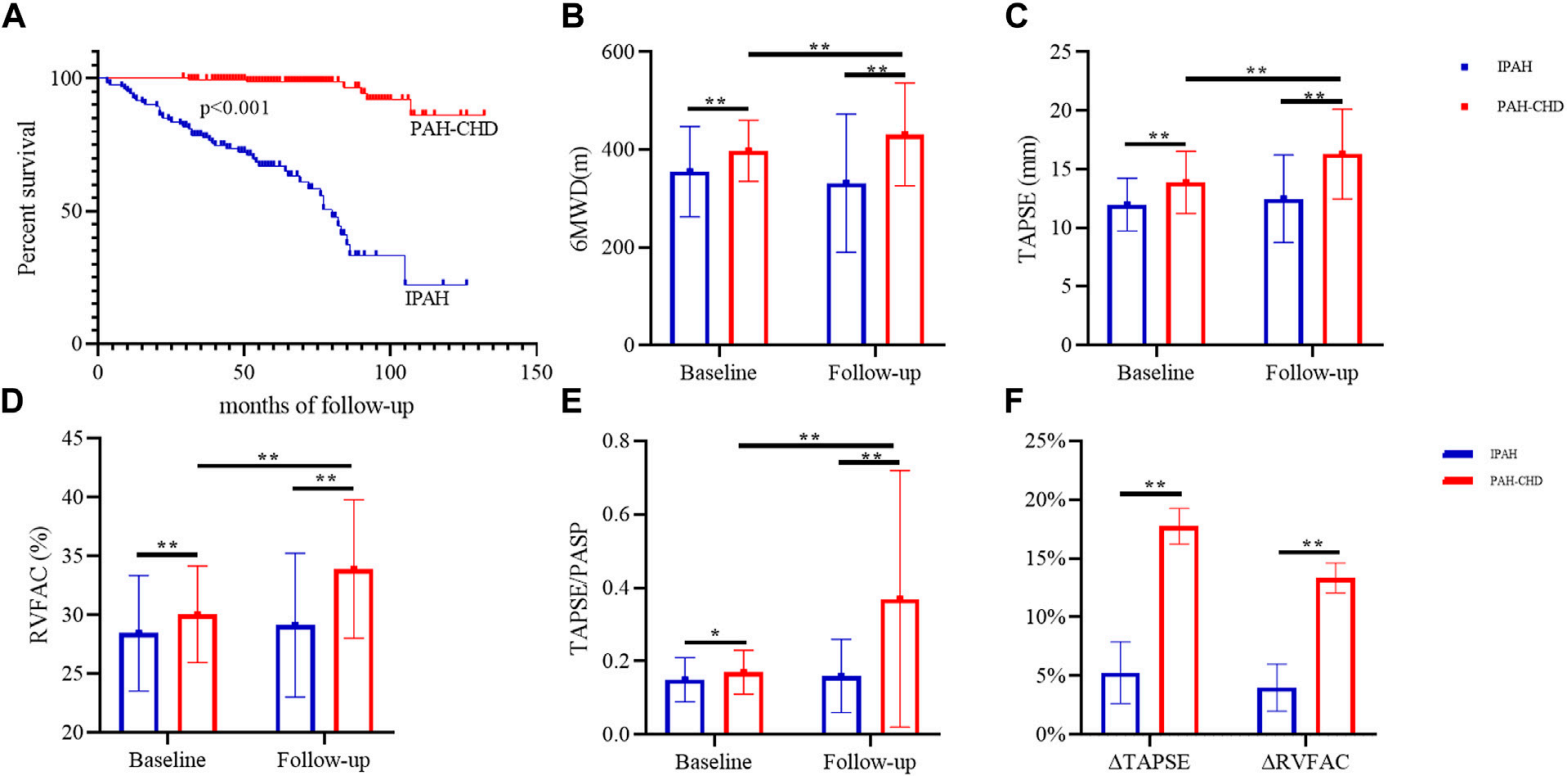
Survival analyses and changes in exercise capacity and RV functional parameters in IPAH and PAH-CHD. Note: **(A)** Kaplan-Meier survival analyses; changes in 6 MWD **(B)**, TAPSE **(C)**, RCFAC **(D)**, and TAPSE/PASP **(E)**; **(F)** the rate of change of TAPSE and RVFAC. *, *p* < 0.01; **, *p* < 0.001. 6 MWD, 6-min walking distance; WHO FC, World Health Organization functional class; TRV, the velocity of tricuspid valve regurgitation; PASP, pulmonary artery systolic pressure; LVEF, left ventricular ejection fraction; TAPSE, tricuspid annular plane systolic excursion; RVFAC, right ventricular fractional area changes.

Treatment strategies for patients with PAH were also evaluated and summarized in Table 1. In terms of treatment strategies, combination therapy and parenteral targeted medication were more common in IPAH. As of the latest follow-up, forty-nine (40.50%) patients with IPAH and six (3.3%) patients with PAH-CHD had died, and two (1.65%) IPAH patients had received lung transplantation. The survival was significantly shorter in patients with IPAH than in patients with PAH-CHD (log-rank *p* < 0.05) (Figure 1). Right heart failure [48 (87.27%)] was the main cause of death. In the remaining seven cases, six (10.91%) patients suffered sudden unexpected death, and one (1.82%) patient died after transthoracic needle biopsy of pulmonary nodules.

Targeted therapy significantly improved WHO FC, PASP, RV-PA coupling and right heart function without affecting left heart function (Table 2; Figure 1). After targeted treatment, 189 (62.38%) patients WHO FC reaches I or II, PASP decreased from 90.74 ± 25.96 to 77.50 ± 32.96 mmHg, TAPSE increases from 13.12 ± 2.67 to 14.77 ± 4.23 mm, RVFAC increased from 29.42 ± 4.51 to 32.01 ± 6.42 mmHg, and TAPSE/RVSP increases from 0.16 ± 0.06 to 0.29 ± 0.30. Remarkably, despite the 6 MWD after targeted treatment increased from 380.44 to 391.54 m, the difference was not statistically significant. Further subgroup analysis demonstrated that these improvements were primarily in patients with PAH-CHD rather than IPAH patients. In PAH-CHD group, 6 MWD increased from 397.75 ± 62.73 to 431.33 ± 104.85 m, TAPSE increased from 13.88 ± 2.66 to 16.30 ± 3.83 mm, PASP decreased from 91.18 ± 25.59 to 69.08 ± 33.76 mmHg, RVFAC increased from 30.06% ± 4.11% to 33.91% ± 5.90%, and TAPSE/PASP increased from 0.17 ± 0.06 to 0.37 ± 0.35.131 (71.98%) patients WHO FC reached I-II, the number of patients with pericardial effusion decreased from 22 (12.09%) to 11 (6.04%). Fortunately, thirty-six PAH-CHD patients underwent successful closure by interventional or surgical methods. In patients with IPAH, RV function parameters (such as TAPSE and RVFAC) were slightly increased but did not reach statistical difference.

**TABLE 2:** Changes in WHO FC and right ventricular parameters for IPAH or PAH-CHD.

Variables	Total patients (*n* = 303)		*p*-value	IPAH (*n* = 121)		*p*-value	PAH-CHD (*n* = 182)		*p*-value
	Baseline	Follow-up		Baseline	Follow-up		Baseline	Follow-up	
6 MWD, m	380.80 ± 78.61	391.54 ± 130.08	0.219	355.31 ± 92.32	331.70 ± 141.37	0.125	397.75 ± 62.73	431.33 ± 104.85	<0.001
WHO FC, n (%)			<0.001			0.606			<0.001
Ⅰ-Ⅱ	130 (42.90)	189 (62.38)		54 (44.63)	58 (47.93)		76 (41.76)	131 (71.98)	
Ⅲ-Ⅳ	173 (57.10)	114 (37.62)		67 (55.37)	63 (52.07)		106 (58.24)	51 (28.02)	
TRV, m/s	4.45 ± 0.72	4.02 ± 1.06	<0.001	4.40 ± 0.72	4.43 ± 0.76	0.766	4.48 ± 0.72	3.75 ± 1.14	<0.001
PASP, mmHg*	90.74 ± 25.96	77.50 ± 32.96	<0.001	90.08 ± 26.59	90.17 ± 27.35	0.981	91.18 ± 25.59	69.08 ± 33.76	<0.001
LVEF, %	61.69 ± 7.74	61.17 ± 6.76	0.377	61.36 ± 6.08	61.71 ± 6.10	0.658	61.90 ± 8.68	60.80 ± 7.15	0.188
TAPSE, mm	13.12 ± 2.67	14.77 ± 4.23	<0.001	11.98 ± 2.25	12.48 ± 3.73	0.212	13.88 ± 2.66	16.30 ± 3.83	<0.001
RVFAC, %	29.42 ± 4.51	32.01 ± 6.42	<0.001	28.45 ± 4.92	29.14 ± 6.12	0.330	30.06 ± 4.11	33.91 ± 5.90	<0.001
TAPSE/PASP	0.16 ± 0.06	0.29 ± 0.30	<0.001	0.15 ± 0.06	0.16 ± 0.10	0.133	0.17 ± 0.06	0.37 ± 0.35	<0.001
Pericardial effusion, n (%)	71 (23.43)	68 (22.44)	0.772	49 (40.50)	57 (47.11)	0.300	22 (12.09)	11 (6.04)	0.045

IPAH, idiopathic pulmonary arterial hypertension; PAH-CHD, pulmonary arterial hypertension associated with congenital heart disease; 6 MWD, 6-min walking distance; WHO FC, World Health Organization functional class; TRV, the velocity of tricuspid valve regurgitation; PASP, pulmonary artery systolic pressure; LVEF, left ventricular ejection fraction; TAPSE, tricuspid annular plane systolic excursion; RVFAC, right ventricular fractional area changes.

Considering that the baseline RV function between the two cohorts was not matched, we used TAPSE (cut-off value 12 mm) as a reference to match the RV function of the two cohorts, so as to further analysed the treatment response to targeted drugs in patients with IPAH and PAH-CHD with different baseline RV functions. The clinical characteristics, echocardiographic, and hemodynamic parameters of RV function-matched patients were summarized in Supplementary Table S1. As presented in Supplementary Table S1, patients with IPAH had a higher proportion (44.63%) of severe (i.e., TAPSE<12 mm) RV dysfunction. There were no statistical differences in age, gender, HR, PASP, LVEF, mRVP, and PAWP between IPAH and PAH-CHD under the new classification. Compared with patients with PAH-CHD, patients with IPAH had higher NT-ProBNP and PVR, and lower mPAP and CI. In patients with severe RV dysfunction, no statistical differences were observed in 6 MWD, WHO FC, and TAPSE between IPAH and PAH-CHD. Targeted therapy significantly improved the WHO FC and TAPSE in patients with severe RV dysfunction, whereas there was no statistical difference in Δ6MWD, ΔTAPSE, and ΔRVFAC between IPAH and PAH-CHD (all *p* > 0.05; Supplementary Table S2). For patients with TAPSE≥12 mm, 6 MWD, WHO FC, and TAPSE in the IPAH group were inferior to those in the PAH-CHD group, and targeted drugs significantly improved the WHO FC, PASP, TAPSE, RVFAC, and RV-PA coupling in patients with PAH-CHD, but had little effect on patients with IPAH (Supplementary Table S2). Collectively, compared with IPAH patients, patients with PAH-CHD were more likely to benefit from targeted drugs with greater improvement than those with IPAH.

## 4 Discussion

Right heart failure is a life-threatening complication of PAH and is associated with increased mortality. In the present study, we retrospectively analysed the differences in baseline RV function and response to targeted therapy between patients with IPAH and patients with PAH-CHD admitted to our centre over the past 10 years. First, the average age of these enrolled population was 36.23 years old and female was predominant. The overall female-to-male ratio was 2.37:1, and the ratio in IPAH group was approximately 1.95:1, which was similar to what we observed in COMPERA (Comparative, Prospective Registry of Newly Initiated Therapies for Pulmonary Hypertension) Registry (Hoeper et al., 2013).

The normal right ventricle is thin-walled, low afterload, and high-compliance distinct from the left ventricle. Anatomic and functional characteristics determine that the right ventricle is more capable of accommodating the volume overload rather than pressure overload (Edward et al., 2022). In PAH, as a consequence of persistently elevated PAP, the RV afterload and wall stress are increased resulting in RV hypertrophy, dilation, and dysfunction under suitable conditions, and the severity is affected by the duration of the pressure overload. Interestingly, abundant literature demonstrated that RV structure and function in people lacking cardiopulmonary disease vary with age, gender, race, exercise (Kawut et al., 2011; Cornwell et al., 2020; Thenappan and Weir, 2020; Edward et al., 2022). Therefore, it is easy to understand the divergent responses of RV to PH subtypes with different pathogenesis and pathophysiology. For example, Emami and colleagues found that patients with IPAH had better RV function compared to those with PH secondary to chronic heart failure (PH-HF) (Emami et al., 2020). This study showed that patients with IPAH had higher peak systolic velocity of tricuspid annulus, higher dp/dt, and better RV free wall stiffness, but other RV parameters such as TAPSE and FAC are only slightly increased without reaching statistical significance. In subgroup analysis of PAH, multiple studies have demonstrated that patients with SSc-PAH had worse resting and reserve RV function than patients with IPAH (Overbeek et al., 2008; Tedford et al., 2013; Hsu et al., 2016; Hsu et al., 2018). This RV dysfunction in SSc-PAH was associated with impaired intrinsic myocardial contractility, decreased calcium cycling, higher RV fibrosis and diastolic stiffness, and RV-PA decoupling (Tedford et al., 2013; Hsu et al., 2016; Hsu et al., 2018).

In our study, the mPAP in IPAH group was significantly lower than that in PAH-CHD group, but a higher baseline PVR implied more severe pulmonary vascular disorders in the IPAH cohort. Pericardial effusion, a relatively common complication of PAH, is associated with increased mortality (Fenstad et al., 2013; Shimony et al., 2013; Chandel et al., 2021). In this study, the incidence of pericardial effusion (40.5%) in patients with IPAH was similar to that in Shimony’s study (47.1%), which may be related to RV dysfunction and subsequent increased right atrial filling pressure (Shimony et al., 2013). Subsequent analysis showed that the RV function (TAPSE, RVFAC) in patients with IPAH was significantly inferior to that in patients with PAH-CHD. As discussed above, RV structure and function are subject to multiple factors, and following conditions may account for the difference in RV function at baseline: firstly, the clinical manifestations in patients with IPAH are non-specific with insidious onset, and some patients present with RV dysfunction or even decompensation at initial diagnosis, which is confirmed by the higher proportion of severe RV dysfunction in the IPAH group in our study. Secondly, pathogenesis participates in this difference. Anomalous systemic-to-pulmonary shunt increases volume-load or pressure-load, which plays an important role in the development of PAH-CHD. In the early stage of PAH-CHD, PVR is only moderately elevated. As PAH-CHD progresses, which may take decades, irreversible pulmonary artery lesions such as plexiform lesions and necrotising arteritis appear, leading to loss of pulmonary vascular beds and subsequent progressive elevation of PVR and PAP with bidirectional or reverse shunt (Van der Feen et al., 2017). In the long-term chronic course of PAH-CHD, right ventricle can better adapt to the elevated afterload and maintain better RV function (Giusca et al., 2016; Brida and Gatzoulis, 2018). Whereas, the mechanism of RV dysfunction in IPAH is more mysterious and complex, including genetics, afterload overload, metabolic transformation, myocardial ischemia, and other aspects (van der Bruggen et al., 2016; Sun et al., 2017; Sanz et al., 2019; Sree et al., 2021). Finally, RV histological features contribute to these differences. Adaptive RV remodelling in PAH-CHD (such as ES) patients is characterized by RV hypertrophy, increased RV contractility, lower RV fibrosis and RV diastolic stiffness (Giusca et al., 2016; van der Bruggen et al., 2017). However, the adaptive RV remodelling in IPAH patients is consistent with a combination of RV hypertrophy, increased RV contractility, higher RV fibrosis and RV diastolic stiffness (van der Bruggen et al., 2017). These features suggest that IPAH patients have poor RV adaptation and are more susceptible to deteriorate from RV hypertrophy to RV failure. Notably, patients with PAH-CHD who met operative indications at initial diagnosis were not included in this study, and this design may narrow the actual gap in baseline RV function between the two cohorts.

At present, assessing the severity of PAH patients by multiparameter risk stratification is an important principle for selecting treatment strategies. A large number of literature has demonstrated that PAH-specific medications, especially combined therapy, improve activity tolerance, quality of life, hemodynamics, and RV structure and function in patients with IPAH and patients with PAH-CHD (D’Alto et al., 2020; Fathallah and Krasuski, 2018; Ruopp and Cockrill, 2022; Vonk et al., 2022). Given that vasodilators are expensive, PAH patients often bear a heavy economic burden. Therefore, we dynamically adjusted treatment strategy based on contemporaneous guidelines and economic conditions. In our study, patients with IPAH had a higher proportion of combination therapy and parenteral prostacycline than patients with PAH-CHD. Targeted therapy remarkably improved overall 6 MWD, WHO FC, PASP, RV function, and survival. Further subgroup analysis showed that vasodilator therapy substantially improved hemodynamic status and RV function in patients with PAH-CHD, and 36 patients successfully repaired the defect. However, although survival in IPAH cohort was improved, RV function was only slightly increased and did not have statistical difference, and 6 MWD even witnessed a downward trend, which was inconsistent with previous studies. To our knowledge, targeted agents relieve symptoms by dilating PA and reducing RV afterload. Patients’ response to targeted therapy was influenced by RV remodelling and treatment strategies, which had also been observed in other subtypes of PH. For instance, patients with CTD-PAH had a worse therapeutic response than those with IPAH (Rhee et al., 2015; Argula et al., 2017; Hsu et al., 2018). Goh and colleagues evaluated RV remodelling by cardiac magnetic resonance (CMR) and demonstrated that the high-volume-low-mass (HVLM, i.e., maladaptive RV remodelling) group had a worse prognosis than the low-volume-low-mass (LVLM, i.e., adaptive RV remodelling) group (Goh et al., 2022). After treatment, a higher proportion (73.5%) of LVLM patients remained in this group, and only 17.4% of HVLM patients converted to LVLM. In our study, IPAH patients had more severe baseline RV dysfunction, RV-PA coupling, and pulmonary vascular disorders. Considering that the baseline RV function was not matched between IPAH and PAH-CHD, we matched the RV function and subsequently analysed the patients’ response to targeted drugs. The results indicated that targeted drugs improved WHO FC and TAPSE in patients with severe RV dysfunction (i.e., TAPSE<12 mm), and there was no significant difference in ΔTAPSE and ΔRV FAC between PAH-CHD and IPAH. Notably, for patients with non-severe RV dysfunction (i.e., TAPSE≥12 mm), patients with PAH-CHD had a better response to treatment than patients with IPAH. The limited effect of targeted agents on PA dilation may partially account for the different treatment responses between two cohorts. Additionally, deterioration of treatment-naive PAH patients has progressed over time. Targeted therapy effectively increases the time to clinical deterioration and improves survival in IPAH patients. However, the follow-up period in our study was relatively longer (up to 132 months), it is necessary to further verify whether the improvement in RV function in IPAH patients receiving treated therapy will continue to improve in the next few years.

Lung transplantation is effective in treating end-stage PAH. However, given the difficulty in obtaining donors and long waiting time, patients with a high or intermediate–high risk of death or with an inadequate response to treatment should be considered as lung transplantation candidates earlier. Several small sample studies (Boucly et al., 2021; D’Alto et al., 2020) have proved that triple combination therapy including parenteral prostacyclin significantly increases transplant-free survival and reverses RV remodelling, suggesting that this may be a good transition for critical patients.

This study still had the following limitations. Firstly, this was a single-center retrospective study. During long-term follow-up, patients’ treatment strategies, especially combination therapy, were dynamically adjusted according to contemporaneous guidelines and economic condition, and this study reflected the effectiveness of the overall treatment strategy. Secondly, we chose conventional parameters to evaluate RV function. Emerging evidence demonstrated that CMR was also effective evaluation tools, and TTE combined with these new indicators may have more important value. Finally, the included population was relatively small, and there were large differences between different groups after matching RV function. Prospective studies with larger populations will be required in the future.

In conclusion, we analysed RV function and its response to PAH-targeted therapy between IPAH and PAH-CHD cohorts. Compared with patients with PAH-CHD, patients with IPAH hade worse baseline RV function, unfavourable prognosis, and inadequate response to targeted treatment. Patients with inadequate response to treatment may be considered as candidates for lung transplantation earlier.

## Data Availability

The original contributions presented in the study are included in the article/Supplementary Material, further inquiries can be directed to the corresponding author.

## References

[B1] Argula R. G. Karwa A. Lauer A. Gregg D. Silver R. M. Feghali-Bostwick C. (2017). Differences in right ventricular functional changes during treatment between systemic sclerosis-associated pulmonary arterial hypertension and idiopathic pulmonary arterial hypertension. Ann. Am. Thorac. Soc. 14 (5), 682–689. 10.1513/AnnalsATS.201608-655OC 28282243 PMC5802595

[B2] Boucly A. Savale L. Jaïs X. Bauer F. Bergot E. Bertoletti L. (2021). Association between initial treatment strategy and Long-Term survival in pulmonary arterial hypertension. Am. J. Respir. Crit. Care Med. 204 (7), 842–854. 10.1164/rccm.202009-3698OC 34185620

[B3] Brida M. Gatzoulis M. A. (2018). Pulmonary arterial hypertension in adult congenital heart disease. Heart 104 (19), 1568–1574. 10.1136/heartjnl-2017-312106 29720395

[B4] Chandel A. Verster A. Rahim H. Khangoora V. Nathan S. D. Ahmad K. (2021). Incidence and prognostic significance of pleural effusions in pulmonary arterial hypertension. Pulm. Circ. 11 (2), 20458940211012366. 10.1177/20458940211012366 33996030 PMC8108083

[B5] Chung L. Liu J. Parsons L. Hassoun P. M. McGoon M. Badesch D. B. (2010). Characterization of connective tissue disease-associated pulmonary arterial hypertension from REVEAL: Identifying systemic sclerosis as a unique phenotype. Chest 138 (6), 1383–1394. 10.1378/chest.10-0260 20507945 PMC3621419

[B6] Chung L. Farber H. W. Benza R. Miller D. P. Parsons L. Hassoun P. M. (2014). Unique predictors of mortality in patients with pulmonary arterial hypertension associated with systemic sclerosis in the REVEAL registry. Chest 146 (6), 1494–1504. 10.1378/chest.13-3014 24992469 PMC4251613

[B7] Cornwell W. K. Tran T. Cerbin L. Coe G. Muralidhar A. Hunter K. (2020). New insights into resting and exertional right ventricular performance in the healthy heart through real-time pressure-volume analysis. J. Physiol. 598 (13), 2575–2587. 10.1113/JP279759 32347547

[B8] D'Alonzo G. E. Barst R. J. Ayres S. M. Bergofsky E. H. Brundage B. H. Detre K. M. (1991). Survival in patients with primary pulmonary hypertension. Results from a national prospective registry. Ann. Intern Med. 115 (5), 343–349. 10.7326/0003-4819-115-5-343 1863023

[B9] D'Alto M. Badagliacca R. Argiento P. Romeo E. Farro A. Papa S. (2020). Risk reduction and right heart reverse remodeling by upfront triple combination therapy in pulmonary arterial hypertension. Chest 157 (2), 376–383. 10.1016/j.chest.2019.09.009 31563498

[B10] Edward J. Banchs J. Parker H. Cornwell W. (2022). Right ventricular function across the spectrum of health and disease. Heart, heartjnl-2021-320526. 10.1136/heartjnl-2021-320526 PMC9985748 35641176

[B11] Emami S. Samiei N. Amin A. Taghavi S. Parsaee M. Naderi N. (2020). Differences in right ventricular dysfunction in patients with idiopathic pulmonary hypertension versus secondary pulmonary hypertension. Adv. Respir. Med. 88 (1), 1–5. 10.5603/ARM.2020.0071 32153001

[B12] Farber H. W. Miller D. P. Poms A. D. Badesch D. B. Frost A. E. Muros-Le R. E. (2015). Five-Year outcomes of patients enrolled in the REVEAL Registry. Chest 148 (4), 1043–1054. 10.1378/chest.15-0300 26066077

[B13] Fathallah M. Krasuski R. A. (2018). A multifaceted approach to pulmonary hypertension in adults with congenital heart disease. Prog. Cardiovasc. Dis. 61 (3-4), 320–327. 10.1016/j.pcad.2018.07.017 30031003

[B14] Fenstad E. R. Le R. J. Sinak L. J. Maradit-Kremers H. Ammash N. M. Ayalew A. M. (2013). Pericardial effusions in pulmonary arterial hypertension: Characteristics, prognosis, and role of drainage. Chest 144 (5), 1530–1538. 10.1378/chest.12-3033 23949692

[B15] Galiè N. Humbert M. Vachiery J. L. Gibbs S. Lang I. Torbicki A. (2016). 2015 ESC/ERS guidelines for the diagnosis and treatment of pulmonary hypertension: The joint task force for the diagnosis and treatment of pulmonary hypertension of the European society of cardiology (ESC) and the European respiratory society (ERS): Endorsed by: Association for European paediatric and congenital cardiology (AEPC), international society for heart and lung transplantation (ISHLT). Eur. Heart J. 37 (1), 67–119. 10.1093/eurheartj/ehv317 26320113

[B16] Giusca S. Popa E. Amzulescu M. S. Ghiorghiu I. Coman I. M. Popescu B. A. (2016). Is right ventricular remodeling in pulmonary hypertension dependent on etiology? An echocardiographic study. Echocardiography 33 (4), 546–554. 10.1111/echo.13112 26542101

[B17] Goh Z. M. Balasubramanian N. Alabed S. Dwivedi K. Shahin Y. Rothman A. M. K. (2022). Right ventricular remodelling in pulmonary arterial hypertension predicts treatment response. Heart 108 (17), 1392–1400. 10.1136/heartjnl-2021-320733 35512982 PMC9380507

[B18] Hoeper M. M. Huscher D. Ghofrani H. A. Delcroix M. Distler O. Schweiger C. (2013). Elderly patients diagnosed with idiopathic pulmonary arterial hypertension: Results from the COMPERA registry. Int. J. Cardiol. 168 (2), 871–880. 10.1016/j.ijcard.2012.10.026 23164592

[B19] Hoeper M. M. P. Humbert M. P. Souza R. P. Idrees M. P. Kawut S. M. P. Sliwa-Hahnle K. P. (2016). A global view of pulmonary hypertension. lancet Respir. Med. 4 (4), 306–322. 10.1016/S2213-2600(15)00543-3 26975810

[B20] Hsu S. Houston B. A. Tampakakis E. Bacher A. C. Rhodes P. S. Mathai S. C. (2016). Right ventricular functional reserve in pulmonary arterial hypertension. Circulation 133 (24), 2413–2422. 10.1161/CIRCULATIONAHA.116.022082 27169739 PMC4907868

[B21] Hsu S. Kokkonen-Simon K. M. Kirk J. A. Kolb T. M. Damico R. L. Mathai S. C. (2018). Right ventricular myofilament functional differences in humans with systemic Sclerosis-Associated versus idiopathic pulmonary arterial hypertension. Circulation 137 (22), 2360–2370. 10.1161/CIRCULATIONAHA.117.033147 29352073 PMC5976528

[B22] Humbert M. Kovacs G. Hoeper M. M. Badagliacca R. Berger R. Brida M. (2022). 2022 ESC/ERS Guidelines for the diagnosis and treatment of pulmonary hypertension. Eur. Heart J. 43 (38), 3618–3731. 10.1093/eurheartj/ehac237 36017548

[B23] Kawut S. M. Lima J. A. Barr R. G. Chahal H. Jain A. Tandri H. (2011). Sex and race differences in right ventricular structure and function: The multi-ethnic study of atherosclerosis-right ventricle study. Circulation 123 (22), 2542–2551. 10.1161/CIRCULATIONAHA.110.985515 21646505 PMC3111939

[B24] Manes A. Palazzini M. Leci E. Bacchi R. M. Branzi A. Galiè N. (2014). Current era survival of patients with pulmonary arterial hypertension associated with congenital heart disease: A comparison between clinical subgroups. Eur. Heart J. 35 (11), 716–724. 10.1093/eurheartj/eht072 23455361

[B25] Miranda W. R. Aboulhosn J. A. Hagler D. J. (2022). Catheterization in adults with Congenital Heart disease: A Primer for the Noncongenital Proceduralist. JACC Cardiovasc. Interv. 15 (9), 907–921. 10.1016/j.jcin.2021.12.020 35512915

[B26] Mitchell C. Rahko P. S. Blauwet L. A. Canaday B. Finstuen J. A. Foster M. C. (2019). Guidelines for performing a comprehensive transthoracic echocardiographic examination in adults: Recommendations from the American society of echocardiography. J. Am. Soc. Echocardiogr. 32 (1), 1–64. 10.1016/j.echo.2018.06.004 30282592

[B27] Overbeek M. J. Lankhaar J. W. Westerhof N. Voskuyl A. E. Boonstra A. Bronzwaer J. G. (2008). Right ventricular contractility in systemic sclerosis-associated and idiopathic pulmonary arterial hypertension. Eur. Respir. J. 31 (6), 1160–1166. 10.1183/09031936.00135407 18216049

[B28] Ramjug S. Hussain N. Hurdman J. Billings C. Charalampopoulos A. Elliot C. A. (2017). Idiopathic and systemic sclerosis-associated pulmonary arterial hypertension: A comparison of demographic, hemodynamic, and mri characteristics and outcomes. Chest 152 (1), 92–102. 10.1016/j.chest.2017.02.010 28223154

[B29] Rhee R. L. Gabler N. B. Sangani S. Praestgaard A. Merkel P. A. Kawut S. M. (2015). Comparison of treatment response in idiopathic and connective tissue disease–associated pulmonary arterial hypertension. Am. J. Respir. Crit. Care Med. 192 (9), 1111–1117. 10.1164/rccm.201507-1456OC 26291092 PMC4642205

[B30] Ruopp N. F. Cockrill B. A. (2022). Diagnosis and treatment of pulmonary arterial hypertension: A review. JAMA 327 (14), 1379–1391. the journal of the American Medical Association. 10.1001/jama.2022.4402 35412560

[B31] Sanz J. Sánchez-Quintana D. Bossone E. Bogaard H. J. Naeije R. (2019). Anatomy, function, and dysfunction of the Right Ventricle: JACC state-of-the-art review. J. Am. Coll. Cardiol. 73 (12), 1463–1482. 10.1016/j.jacc.2018.12.076 30922478

[B32] Shimony A. Fox B. D. Langleben D. Rudski L. G. (2013). Incidence and significance of pericardial effusion in patients with pulmonary arterial hypertension. Can. J. Cardiol. 29 (6), 678–682. 10.1016/j.cjca.2012.04.009 22717247

[B33] Sree R. K. Shah R. Stokes M. Walls A. Woodman R. J. Perry R. (2021). Right ventricular myocardial deoxygenation in patients with pulmonary artery hypertension. J. Cardiovasc Magn. Reson 23 (1), 22. 10.1186/s12968-020-00694-0 33678188 PMC7938464

[B34] Sun X. Abbate A. Bogaard H. (2017). Role of cardiac inflammation in right ventricular failure. Cardiovasc. Res. 113 (12), 1441–1452. 10.1093/cvr/cvx159 28957536

[B35] Tedford R. J. Mudd J. O. Girgis R. E. Mathai S. C. Zaiman A. L. Housten-Harris T. (2013). Right ventricular dysfunction in systemic sclerosis-associated pulmonary arterial hypertension. Circ. Heart Fail 6 (5), 953–963. 10.1161/CIRCHEARTFAILURE.112.000008 23797369 PMC3815697

[B36] Tello K. Wan J. Dalmer A. Vanderpool R. Ghofrani H. A. Naeije R. (2019). Validation of the tricuspid annular plane systolic Excursion/Systolic pulmonary artery pressure ratio for the assessment of right Ventricular-Arterial coupling in severe pulmonary hypertension. Circ. Cardiovasc Imaging 12 (9), e009047. 10.1161/CIRCIMAGING.119.009047 31500448 PMC7099862

[B37] Thenappan T. Weir E. K. (2020). Pulmonary arterial hypertension and sex in the right ventricle: It is an interesting picture. Am. J. Respir. Crit. Care Med. 202 (7), 928–929. 10.1164/rccm.202006-2147ED 32640166 PMC7528806

[B38] van der Bruggen C. E. Happé C. M. Dorfmüller P. Trip P. Spruijt O. A. Rol N. (2016). Bone morphogenetic protein receptor type 2 mutation in pulmonary arterial hypertension: A view on the right ventricle. Circulation 133 (18), 1747–1760. 10.1161/CIRCULATIONAHA.115.020696 26984938

[B39] van der Bruggen C. E. E. Tedford R. J. Handoko M. L. van der Velden J. de Man F. S. (2017). RV pressure overload: From hypertrophy to failure. Cardiovasc. Res. 113 (12), 1423–1432. 10.1093/cvr/cvx145 28957530

[B40] van der Feen D. E. Bartelds B. de Boer R. A. Berger R. (2017). Pulmonary arterial hypertension in congenital heart disease: Translational opportunities to study the reversibility of pulmonary vascular disease. Eur. Heart J. 38 (26), 2034–2041. 10.1093/eurheartj/ehx034 28369399

[B41] Vonk N. A. Channick R. Cottreel E. Kiely D. G. Marcus J. T. Martin N. (2022). The REPAIR study: Effects of macitentan on RV structure and function in pulmonary arterial hypertension. JACC Cardiovasc Imaging 15 (2), 240–253. 10.1016/j.jcmg.2021.07.027 34801462

