# Supplementary material for: Differences in right ventricular function and response to targeted therapy between patients with IPAH and PAH-CHD
Source: Front Pharmacol. 2023 Feb 13;14:1124746. doi: 10.3389/fphar.2023.1124746 (PMC9968930; doi:10.3389/fphar.2023.1124746)
Supplement: Supplementary file 1 [file Table2.DOCX]

**Supplemental table 1 clinical characteristics of** **functionally-matched patients with IPAH and PAH-CHD**

| **Variables** | **TAPSE <12mm** | |  | **TAPSE ≥12mm** | |  |
| --- | --- | --- | --- | --- | --- | --- |
|  | **IPAH (n=54)** | **PAH-CHD (n=34)** | **P value** | **IPAH(n=67)** | **PAH-CHD (n=148)** | **P value** |
| Female sex, n (%) | 37 (68.52) | 25 (73.53) | 0.616 | 43 (64.18) | 108 (72.97) | 0.192 |
| Age, y | 38.26±14.54 | 35.26±13.94 | 0.297 | 37.39±12.25 | 35.19±12.74 | 0.255 |
| HR, bpm | 88.33±14.38 | 87.65±16.15 | 0.821 | 83.46±12.41 | 84.49±13.71 | 0.614 |
| SaO_2_ | 94.71±3.22 | 89.57±5.37 | <0.001 | 95.50±2.81 | 93.91±3.72 | 0.004 |
| NT-ProBNP, ng/ml | 3001 (1515.75, 5815.5) | 641.5 (152.25, 2012.75) | <0.001 | 1437(565, 2422) | 713.5 (234.75, 1380.5) | 0.001 |
| 6MWD, m | 311.09±109.03 | 335.65±62.29 | 0.100 | 390.94±55.34 | 412.01±53.65 | 0.036 |
| WHO FC, n (%) |  |  | 0.173 |  |  | 0.032 |
| Ⅰ-Ⅱ | 10 (18.52) | 2 (5.88) |  | 44 (65.67) | 74 (50) |  |
| Ⅲ-Ⅳ | 44 (81.48) | 32 (94.12) |  | 23 (34.33) | 74 (50) |  |
| Echocardiography |  |  |  |  |  |  |
| TRV, m/s | 4.53±0.72 | 4.61±0.95 | 0.640 | 4.30±0.72 | 4.45±0.66 | 0.162 |
| PASP, mmHg* | 95.44±27.42 | 93.29±35.26 | 0.705 | 85.76±25.83 | 90.69±22.94 | 0.197 |
| LVEF, % | 61.11±6.67 | 60.35±6.88 | 0.655 | 61.57±5.60 | 62.26±9.03 | 0.546 |
| TAPSE, mm | 9.94±1.18 | 10.34±0.92 | 0.306 | 13.63±1.39 | 14.70±2.23 | < 0.001 |
| RVFAC, % | 25.45±4.28 | 27.21±4.96 | 0.045 | 30.86±4.00 | 30.72±3.60 | 0.811 |
| TAPSE/PASP | 0.11±0.04 | 0.13±0.06 | 0.164 | 0.17±0.06 | 0.18±0.06 | 0.955 |
| Right heart catheterization |  |  |  |  |  |  |
| mPAP, mmHg | 60.87±12.10 | 74.71±14.61 | < 0.001 | 56.24±12.64 | 65.25±17.40 | < 0.001 |
| mRVP, mmHg | 43.37±9.27 | 48.21±12.27 | 0.060 | 40.48±14.22 | 42.74±11.11 | 0.191 |
| mRAP, mmHg | 13.31±7.32 | 11.41±8.88 | 0.148 | 12.63±5.04 | 9.89±4.98 | 0.002 |
| PAWP, mmHg | 8.85±1.66 | 9.71±2.33 | 0.059 | 8.31±1.74 | 8.42±2.34 | 0.728 |
| Qp, L/min | 2.63±0.91 | 4.08±1.71 | < 0.001 | 3.67±1.15 | 5.56±1.73 | < 0.001 |
| Qs, L/min | 2.63±0.91 | 4.43±2.23 | < 0.001 | 3.67±1.15 | 3.87±1.51 | 0.341 |
| Qp/Qs | 1 | 1.07±0.61 | 0.429 | 1 | 1.54±0.54 | < 0.001 |
| CI, L/min/m2 | 1.76±0.55 | 2.87±1.12 | < 0.001 | 2.36±0.74 | 3.90±1.26 | < 0.001 |
| PVR, WU | 21.81±8.01 | 18.80±8.21 | 0.034 | 14.36±6.05 | 11.40±5.55 | 0.002 |
| SvO_2_, % | 53.30±10.17 | 61.41±7.94 | < 0.001 | 61.73±7.92 | 62.87±8.13 | 0.338 |

**Note:** PAH, pulmonary arterial hypertension; IPAH, idiopathic PAH; CHD, congenital heart disease; PAH-CHD, PAH associated with CHD; HR, heart rate; bpm, beats/min; NT-proBNP, N-terminal pro hormone brain natriuretic peptide; 6MWD, 6-minute walking distance; WHO FC, World Health Organization functional class; TRV, the velocity of tricuspid valve regurgitation; PASP, pulmonary artery systolic pressure; LVEF, left ventricular ejection fraction; TAPSE, tricuspid annular plane systolic excursion; RVFAC, right ventricular fractional area changes; mPAP, mean pulmonary artery pressure; mRVP, mean right ventricular pressure; mRAP, mean right atrial pressure; PAWP, pulmonary artery wedge pressure; Qp, pulmonary artery blood flow; Qs, systemic artery blood flow; CI, cardiac index; PVR, pulmonary vascular resistance; WU, Wood unites; SvO_2_, oxygen saturation of mixed venous blood.

**Supplemental table 2 Changes in right ventricular parameters in functionally-matched patients with IPAH and PAH-CHD**

| **Variables** | **TAPSE <12mm** | |  | **TAPSE ≥12mm** | |  |
| --- | --- | --- | --- | --- | --- | --- |
|  | **IPAH (n=54)** | **PAH-CHD (n=34)** | **P value** | **IPAH(n=67)** | **PAH-CHD (n=148)** | **P value** |
| 6MWD, m | 307.43±152.16 | 357.35±106.60 | 0.052 | 351.27±129.91 | 448.32±97.10 | < 0.001 |
| Δ6MWD, % | 1.61±43.15 | 10.44±40.07 | 0.340 | -9.85±33.20 | 8.72±21.28 | < 0.001 |
| WHO FC, n (%) |  |  | 0.158 |  |  | < 0.001 |
| Ⅰ-Ⅱ | 24 (44.44) | 10 (29.41) |  | 34 (50.75) | 121 (81.76) |  |
| Ⅲ-Ⅳ | 30 (55.56) | 24 (70.59) |  | 33 (49.25) | 27 (18.24) |  |
| TRV, m/s | 4.44±0.67 | 4.02±1.09 | 0.055 | 4.42±0.83 | 3.69±1.14 | < 0.001 |
| PASP, mmHg* | 90.33±24.34 | 76.62±32.58 | 0.047 | 90.03±29.73 | 67.34±33.90 | < 0.001 |
| LVEF, % | 63.06±6.27 | 61.65±5.30 | 0.339 | 60.63±5.78 | 60.61±7.51 | 0.985 |
| TAPSE, mm | 11.30±3.47 | 12.42±2.97 | 0.141 | 13.42±3.68 | 17.20±3.44 | < 0.001 |
| ΔTAPSE, % | 13.59±30.85 | 20.56±29.58 | 0.298 | -1.41±25.62 | 17.09±17.92 | < 0.001 |
| RVFAC, % | 27.49±5.83 | 29.54±5.95 | 0.102 | 30.47±6.06 | 34.91±5.42 | < 0.001 |
| ΔRVFAC, % | 10.00±25.95 | 9.63±18.39 | 0.943 | -0.83±17.44 | 14.20±16.74 | < 0.001 |
| TAPSE/PASP, mm/mmHg | 0.10±0.05 | 0.96±0.35 | < 0.001 | 0.21±0.11 | 0.23±0.15 | 0.400 |
| ΔTAPSE/PASP, % | 2.41±64.28 | 792.57±492.05 | < 0.001 | 39.44±89.87 | 49.94±115.0 | 0.509 |

**Note:** IPAH, idiopathic pulmonary arterial hypertension; PAH-CHD, pulmonary arterial hypertension associated with congenital heart disease; 6MWD, 6-minute walking distance; WHO FC, World Health Organization functional class; TRV, the velocity of tricuspid valve regurgitation; PASP, pulmonary artery systolic pressure; LVEF, left ventricular ejection fraction; TAPSE, tricuspid annular plane systolic excursion; RVFAC, right ventricular fractional area changes.
