# Supplementary material for: Differences in right ventricular function and response to targeted therapy between patients with IPAH and PAH-CHD
Source: Front Pharmacol. 2023 Feb 13;14:1124746. doi: 10.3389/fphar.2023.1124746 (PMC9968930; doi:10.3389/fphar.2023.1124746)
Supplement: Supplementary file 2 [file Table3.DOCX]

**Supplemental table 2 Changes in right ventricular parameters in functionally-matched patients with IPAH and PAH-CHD**

| **Variables** | **TAPSE <12mm** | |  | **TAPSE ≥12mm** | |  |
| --- | --- | --- | --- | --- | --- | --- |
|  | **IPAH (n=54)** | **PAH-CHD (n=34)** | **P value** | **IPAH(n=67)** | **PAH-CHD (n=148)** | **P value** |
| 6MWD, m | 307.43±152.16 | 357.35±106.60 | 0.052 | 351.27±129.91 | 448.32±97.10 | < 0.001 |
| Δ6MWD, % | 1.61±43.15 | 10.44±40.07 | 0.340 | -9.85±33.20 | 8.72±21.28 | < 0.001 |
| WHO FC, n (%) |  |  | 0.158 |  |  | < 0.001 |
| Ⅰ-Ⅱ | 24 (44.44) | 10 (29.41) |  | 34 (50.75) | 121 (81.76) |  |
| Ⅲ-Ⅳ | 30 (55.56) | 24 (70.59) |  | 33 (49.25) | 27 (18.24) |  |
| TRV, m/s | 4.44±0.67 | 4.02±1.09 | 0.055 | 4.42±0.83 | 3.69±1.14 | < 0.001 |
| PASP, mmHg* | 90.33±24.34 | 76.62±32.58 | 0.047 | 90.03±29.73 | 67.34±33.90 | < 0.001 |
| LVEF, % | 63.06±6.27 | 61.65±5.30 | 0.339 | 60.63±5.78 | 60.61±7.51 | 0.985 |
| TAPSE, mm | 11.30±3.47 | 12.42±2.97 | 0.141 | 13.42±3.68 | 17.20±3.44 | < 0.001 |
| ΔTAPSE, % | 13.59±30.85 | 20.56±29.58 | 0.298 | -1.41±25.62 | 17.09±17.92 | < 0.001 |
| RVFAC, % | 27.49±5.83 | 29.54±5.95 | 0.102 | 30.47±6.06 | 34.91±5.42 | < 0.001 |
| ΔRVFAC, % | 10.00±25.95 | 9.63±18.39 | 0.943 | -0.83±17.44 | 14.20±16.74 | < 0.001 |
| TAPSE/PASP, mm/mmHg | 0.10±0.05 | 0.96±0.35 | < 0.001 | 0.21±0.11 | 0.23±0.15 | 0.400 |
| ΔTAPSE/PASP, % | 2.41±64.28 | 792.57±492.05 | < 0.001 | 39.44±89.87 | 49.94±115.0 | 0.509 |

**Note:** IPAH, idiopathic pulmonary arterial hypertension; PAH-CHD, pulmonary arterial hypertension associated with congenital heart disease; 6MWD, 6-minute walking distance; WHO FC, World Health Organization functional class; TRV, the velocity of tricuspid valve regurgitation; PASP, pulmonary artery systolic pressure; LVEF, left ventricular ejection fraction; TAPSE, tricuspid annular plane systolic excursion; RVFAC, right ventricular fractional area changes.
